# Study on the pro-inflammatory mechanism of the HuD antibody in promoting M1 polarization and paraneoplastic neurological syndrome occurrence

**DOI:** 10.1080/21655979.2022.2051267

**Published:** 2022-03-16

**Authors:** Liang Yin, Wen-Ling Yuan, Ke Wu, Li-Na Zhang, Qian-Qian Li

**Affiliations:** Department of Neurology, The First Affiliated Hospital of Bengbu Medical College, Bengbu, Bengbu Anhui, China

**Keywords:** Paraneoplastic neurological syndrome, neuronal cells, macrophages, inflammatory factors

## Abstract

Paraneoplastic neurological syndrome (PNS) is a nonmetastatic complication of malignant tumors that may lead to immune-mediated neuronal dysfunction or death. The occurrence of PNS results from the binding of anti-neuronal antibodies to neuronal cell surface antigens or intracellular antigens, which hinders the function of target proteins and promotes cell death. The aim of this study is to research the effect and immune mechanism of the neuronal ELAV-like protein (HuD antibody) on PNS-related syndrome. Neuronal cells were co-cultured with monocyte macrophages with or without HuD antibody. Next, we detected the apoptosis of neuronal cells by flow cytometry. Meantime, macrophage M1/M2 polarization factors and the secretion of inflammatory factors in the co-culture system were also detected by quantitative polymerase chain reaction (qPCR), Western blots and ELISA technologies. The results showed that after adding the HuD antibody in the co-culture system, the apoptosis level of the neuroma cells were significantly increased, and the apoptosis level were not significant changed when co-culture with monocytes without HuD antibody. In addition, the level of factors of M1 macrophages TNF-α, IL-12, TGF-β and IFN-γ increased, while the level of factors of M2 macrophages IL-10, IL-4, and Arg-1 decreased. The outcomes demonstrated that absorption of the HuD antibody by cerebellar neuronal cells could promote the proliferation of M1 macrophages and stimulates macrophages to secrete inflammatory factors, further damage the neuronal cells, eventually resulting in the occurrence of PNS. This finding provided a theoretical basis for the subsequent treatment and prevention of PNS.

## Graphical Abstract

**Figure:**
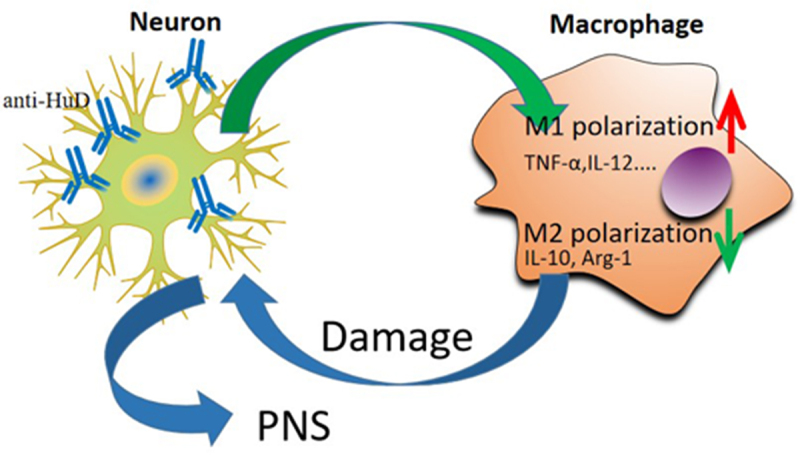

## Introduction

Paraneoplastic neurological syndrome (PNS) is a disease that occurs in some patients with malignant tumors. It affects the distant nervous system and causes nervous system dysfunction when the tumor does not metastasize. For example, lung cancer and ovarian cancer can present the distant effect of gray matter inflammation and neurodegeneration of the central nervous system (CNS) [1,2]. The occurrence of PNS results from the binding of anti-neuronal antibodies to neuronal cell surface antigens or intracellular antigens, hindering the function of target proteins and promoting cell death. Typical paraneoplastic neurological phenotypes include subacute cerebellar degeneration, marginal encephalitis, encephalomyelitis, and back sensory neuropathy [3]. In the clinical manifestations of many patients, it is found that specific antibodies are usually associated with some types of tumor [4]. Detecting patients’ autoantibodies can help diagnose PNS and provide the first evidence of potential tumors, which is very important for the screening of malignant tumors. Antibody-mediated immunotherapy also plays an important role in the treatment of patients with paraneoplastic neurological diseases, and cell surface antibodies are usually more effective than intracellular antibodies. Therefore, potential tumors can be removed by paraneoplastic antibodies [5].

Subacute sensory neuropathy/encephalomyelopathy syndrome (Hu syndrome) is characterized by a high-titer antibody response to the tumor nerve antigen HuD (also known as ELAVL4), which is usually expressed only in neurons and small cell lung cancer (SCLC) [6]. In the serum and cerebrospinal fluid (CSF) of patients with PNS, the high-titer antibody of the neuronal antigen HuD provides preliminary evidence for the immunological basis of PNS. The patient’s antiserum can also identify the HuD antigen, which can facilitate the regulation of tumor mRNA stability and the translation process of RNA binding protein [7]. In patients with SCLC, HuD neuronal antigen expression is considered to promote immune surveillance and inhibit tumor growth. Accompanied by spontaneous tumor regression in a few patients, this indicates that a HuD-mediated immune response may be helpful for the cure of tumors in patients [8]. Some studies have revealed that tumor cells expose the expressed HuD antigen to the immune system to produce a HuD-specific immune response. Patients with Hu syndrome usually present with clinical neurological symptoms, which are triggered by a weakening of the tumor immune response in the nervous system [9]. However, a study revealed that the HuD expression in patients with Hu syndrome could trigger PNS [10], but the specific mechanism remains unclear [11]. We supposed that the HuD antibody free in peripheral blood could be absorbed by neurocyte and further stimulate the macrophage M1 polarization in the immune microenvironment, which eventually resulted in the occurrence of PNS.

This study revealed the effect of the HuD antibody on neuroma cell apoptosis and the role of the HuD antibody in the pathogenesis of PNS in vitro, aiming to provide the theoretical basis for immunotherapy and diagnosis of PNS.

## Materials and methods

### Test materials and reagents

HuD antibody was purchased from Abcam (Shanghai) Trading Co., Ltd. Human neuroepithelioma-derived cell SK-N-MC and human monocyte U937 were purchased from Ningbo Mingzhou Biotechnology Co., Ltd. The cell test groups are as follows: HuD antibody + human neuron cell + U937 cell for co-culture; HuD antibody + human neural cell for co-culture; human neural cells + U937 cells for co-culture; human nerve cells. SK-N-MC cells were grown in MEM, supplemented with 10% fetal bovine serum, penicillin (100 U/mL) and streptomycin (100 mg/ml) in 24-well tissue culture plates. U937 cells were maintained in RPMI 1640 medium supplemented with 10% FBS. Transwell cell culture system was used to co-culture the two cells. According the previous methods [12] in brief, SK-N-MC cells were plated on the bottom of the six-well transwell cell culture system, while U937 cells were cultured onto the membrane of transwell cell culture inserts.

### Experimental methods

#### Flow cytometry analysis

Neuroma cells were digested with ethylenediaminetetraacetic acid (EDTA)-free 0.25% trypsin. Digestion was terminated, and the cells were collected and centrifuged at 1,500 rpm for 5 min. The supernatant was discarded, and the cells were collected. According to the instructions of the ANNEXIN V- FITC/PI Kit (CA1020, Solarbio), the cells were washed with cold phosphate buffered saline (PBS) and centrifuged at 1,500 rpm for 5 min. After adding 300 µL of binding buffer, the cells were resuspended, and then 5 µL of the V-fluorescein isothiocyanate (FITC) antibody was added. The mixture was incubated at room temperature in a dark for 15 min. After that, propidium iodide (PI) antibody was added to the mixture then gently mixed and cultured in a dark at room temperature for 105 min. Apoptosis was detected by flow cytometry and analyzed by the Cell Quest software.

#### Enzyme-linked immunosorbent assay (ELISA)

The expressions of inflammatory factors were detected by using an ELISA Kit (Buya-TEK, China), and the washing solution was diluted 20 times with sterile distilled water to the original washing solution. The enzyme-labeled antigen (50 µL) was added to a 96-well plate along with the sample (50 µL), using PBS as the control. Then, the 96-well plate was incubated in a water bath or an incubator at 37°C for 30 minutes. After culture, developer was added to the sample. The absorbance was measured at 450 nm using a microplate reader (Thermo Fisher Scientific, USA).

#### Quantitative polymerase chain reaction (qPCR) analysis

Total cellular RNA was extracted using a TRIzol reagent (Invitrogen, California, USA), and quantified by NanoDrop spectrophotometer. The cDNA was synthesized using 1 μg total RNA and a TaKaRa RNA PCR kit. The expression levels of polarization and inflammatory factors in each group were detected by using an RT-PCR Kit (Invitrogen, California, USA). The PCR reaction conditions were 95°C for 5 min followed by 40 cycles at 95°C for 15 s and 60°C for 30 s in 20 μl reaction system. The relative quantification of RNA expression was performed using the 2^−ΔΔCT^ method. The primers were shown as follows: IL-10, Forward (F): CGGGAAGACAATAACTGCACCC; Reverse (R): CGGTTAGCAGTATGTTGTCCAGC. Arg-1, F: CTCCAAGCCAAAGTCCTTAGAG; AGGAGCTGTCATTAGGGACATC. TNF-α, F: ATTGCCCTGTGAGGAGGAC; R: TGAGCCAGAAGAGGTTGAGG. TGF-β, F: AACATGATCGTGCGCTCTGCAAGTGCAGC; R: AAGGAATAGTGCAGACAGGCAGGA. IL-4, F: CCGTAACAGACATCTTTGCTGCC; R: GAGTGTCCTTCTCATGGTGGCT. IFN-γ, F: AGCTCTGCATCGTTTTGGGTT; R: GTTCCATTATCCGCTACATCTGAA. IL-1β, F: CACCTCAAGAACATCCAGAGCT; CAAGCAGAACTGAACTACCATCG. IL-12, F: TGCCTTCACCACTCCCAAAACC; R: CAATCTCTTCAGAAGTGCAAGGG. GAPDH, F: TCTGGCACCACACCTTCTA; R: AGGCATACAGGGACAGCAC.

#### Western Blot (WB)

The cells were lysed in a radio-immunoprecipitation assay (RIPA) buffer and centrifuged at 12,000 *g* and 4°C for 10 minutes. The total protein was extracted and the protein samples were separated using sodium dodecyl sulfate-polyacrylamide gel electrophoresis (SDS-PAGE). The protein was then transferred to a polyvinylidene fluoride (PVDF) film, and, subsequently, the membrane was blocked in 5% skimmed milk at room temperature for one hour. A primary antibody was then added at the ratio of 1:2000 and incubated overnight at 4°C. The next day, the membrane was washed three times with Tris-buffered saline containing Tween 20 (TBST) and incubated with a horseradish peroxidase (HRP)-labeled sheep anti-mouse secondary antibody for 1 hour at room temperature. Finally, enhanced chemiluminescence (ECL) substrate (Applygen, Beijing, China) was used to observe protein expression.

Statistical analysis

SPSS 19.0 and GraphPad Prism 8.0 software were used to data statistics and graphing. Data are shown as mean ± standard deviation (Mean ± SD). Student *T* test was used to compare the statistical differences between the two groups, while one-way ANOVA (and nonparametric) test was used to assess the parameters among the three groups. *P* value <0.05 was considered as a significant difference.

## Results

In the peripheral blood, HuD antibody participated in the immune response and promoted the occurrence of PNS. We think that the HuD antibody can be absorbed by nerve cells and further promote the M1 polarization of macrophage, which secreted the inflammatory factors and induced the apoptosis of nerve cells. To test our hypothesis, we co-culture the nerve cells and monocyte cell with or without the HuD antibody, and analysis the level of macrophage polarization and neuronal apoptosis.

### Effect of the HuD antibody on neurocyte apoptosis and macrophage-related inflammatory factor expression

**Figure 1.**
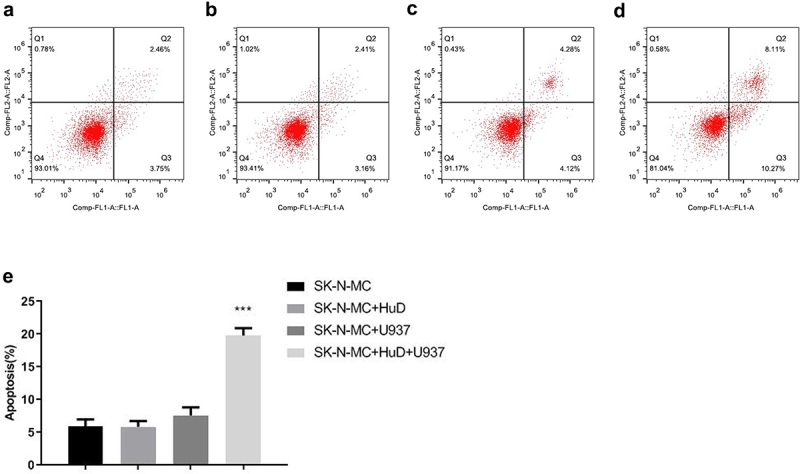
The apoptosis levels of co-cultured neuroma cells and monocytes in the presence of HuD proteins by flow cytometry are shown. (a) Neuroma cells. (b) Addition of HuD proteins into neuroma cells. (c) Co-cultured neuroma cells and monocytes. (d) Co-cultured neuroma cells and monocytes in the presence of the Hud protein. (e) Quantitative analysis of apoptosis by flow cytometry (‘***’ represents an extremely significant difference [*P* < 0.001]; ‘**’ represents a significant difference [*P* < 0.01]; ‘*’ represents a statistical difference [*P* < 0.05]; “ns“ means no significant difference).

The apoptosis image and quantitative analysis results of flow cytometry indicate that after adding the HuD antibody, the apoptosis level of neuroma cells co-cultured with monocytes increased significantly (*P* < 0.001), but when neuroma cells were not co-cultured with monocytes, the apoptosis level of neuroma cells did not change significantly after adding the HuD antibody (Figure 1). This suggests that neuroma cells may stimulate macrophages to secrete inflammatory factors and induce neuroma cell apoptosis under the action of the HuD protein.

### Detection of PNS-related inflammatory factors secreted by macrophages mediated by the HuD antibody

**Figure 2.**
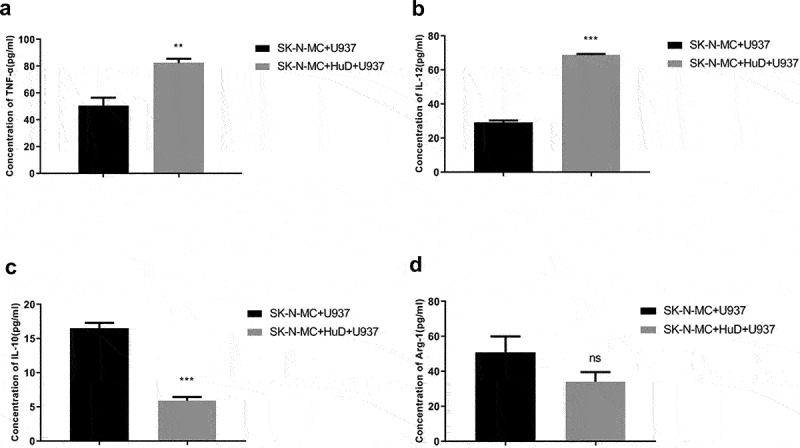
Detection of different concentrations of paraneoplastic neurological syndrome (PNS)-associated inflammatory cytokines is shown. (a) Detection of TNF-α concentration. (b) Detection of IL-12 concentration. (c) Detection of IL-10 concentration. (d) Detection of Arg-1 concentration (‘***’ represents an extremely significant difference [*P* < 0.001]; ‘**’ represents a significant difference [*P* < 0.01]; ‘*’ represents a statistical difference [*P* < 0.05]; ‘ns’ means no significant difference).

The concentrations of PNS-related inflammatory factors secreted by macrophages were detected by ELISA. As shown in Figure 2, compared with the non-HuD antibody group, factors secreted by M1 macrophages, such as TNF-α (*P* < 0.01) and IL-12 (*P* < 0.001), increased significantly in the HuD antibody group; contrarily, the concentrations of factors secreted by M2 macrophages, such as IL-10 (*P* < 0.001), decreased significantly. This suggests that neuroma cells stimulated by the HuD antibody have an effect on the polarization of macrophages, inducing polarization toward the M1 pro-inflammatory direction. Compared with the non-HUD antibody group, the concentration of TNF-α induced by M1 macrophages increased, but the concentration of Arg-1 induced by M2 macrophages decreased significantly (*P* < 0.01), exacerbating inflammatory damage to cells. Other inflammatory factors also presented with corresponding changes during the process. In some cases, no significant difference was detected. This further suggests that neuroma cells treated by the HuD antibody stimulate macrophages to undergo polarization toward the M1 phenotype.

### The mRNA expression level of PNS-related inflammatory factors

**Figure 3.**
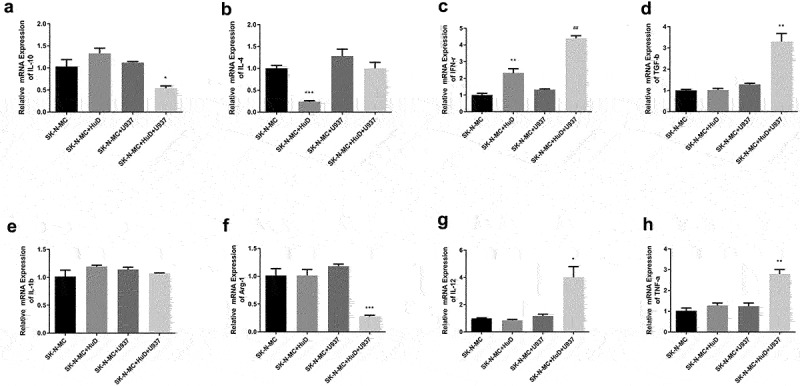
The mRNA expression levels of PNS-associated inflammatory factors are shown. (a) Detection of IL-10 expression level. (b) Detection of IL-4 expression level. (c) Detection of IFN-γ expression level. (d) Detection of TGF-β expression level. (e) Detection of IL-1β expression level detected. (f) Detection of Arg-1 expression level. (g) Detection of IL-12 expression level. (h) Detection of TNF-α expression level (‘***’ represents an extremely significant difference [*P* < 0.001]; ‘**’ represents a significant difference [*P* < 0.01]; ‘*’ represents a statistical difference [*P* < 0.05]; ‘ns’ means no significant difference).

The expressions of cell polarization factors and the inflammatory factor mRNA were detected by conducting a qPCR test. After adding the HUD antibody, the levels of factors secreted by M1 macrophages TNF-α (*P* < 0.01) and IL-12 (*P* < 0.05) in co-cultured neuroma cells and monocyte macrophages increased significantly, while the level of factor secreted by M2 macrophage IL-10 (P < 0.05) decreased (Figure 3). This suggests that the HuD antibody induces macrophages to polarize in the M1 direction and promotes neuroma cell injury through its own pro-inflammatory effect. Compared with neuroma cell cultured alone, secretions of M1 macrophage-inducible factors, such as IFN-γ and TGF-ß, were increased after adding the HuD protein, while the secretions of M2 macrophage-inducible factors, such as IL-4 and Arg-1, were decreased. This further suggests the effect of neuroma cells absorbing HuD proteins on macrophage polarization. Other inflammatory factors also underwent corresponding changes during the process, but a significant difference was not detected in some cases.

### Effect of the HuD antibody on polarization of M1 and M2 macrophages

**Figure 4.**
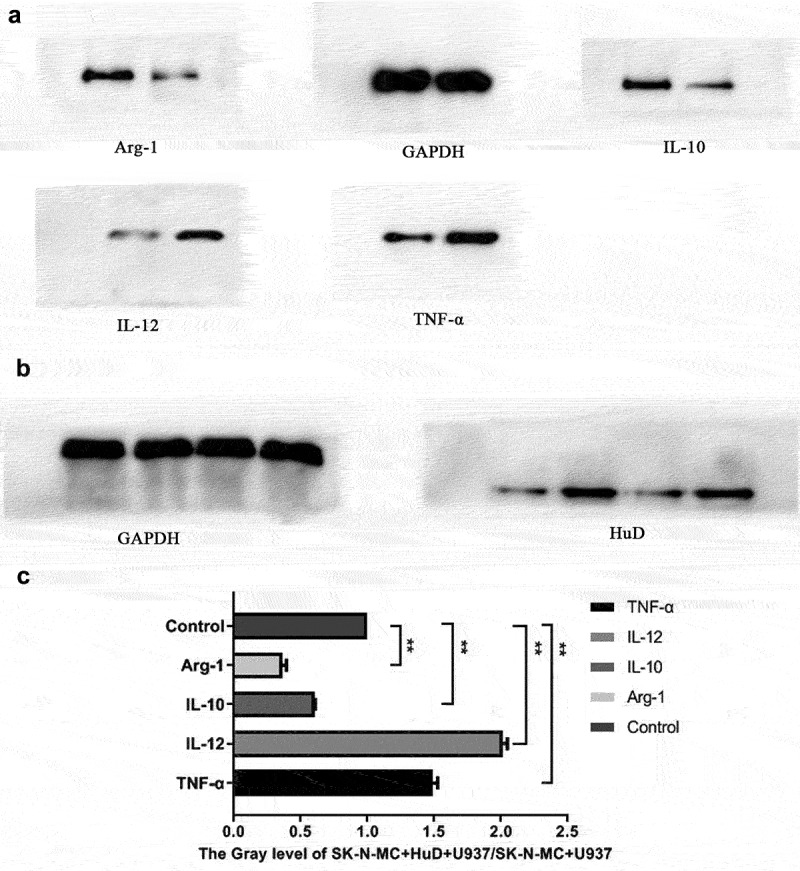
Detection of changes in macrophage polarization factor levels is shown.

After co-culture of the neuroma cells and macrophages (shown in Figure 4a), the expression levels of factors secreted by macrophages were detected in the presence or absence of the HuD antibody. Figure 4a shows changes in macrophage polarization factor levels. As shown in Figure 4b, Western blot results revealed that the concentration in the HuD antibody group was significantly higher than that in the non-HuD antibody group and the expression of the HuD protein in the SK-N-MC + HuD + U937 group was higher than that in the SK-N-MC + U937 group. As shown in Figure 4c, when neuroma cells were cultured with the HuD antibody, factors secreted by M1 macrophages (TNF-α and IL-12) increased significantly, and factors secreted by M2 phenotypes (IL-10 and Arg-1) decreased significantly (P < 0.01).

(a) Changes in levels of TNF-α, IL-12, IL-10 and Arg-1 secreted factors in the presence or absence of HuD proteins; glyceraldehyde 3-phosphate dehydrogenase (GAPDH) is used as internal control. (b) Detection of the content of HuD histone in each group; GAPDH is used as internal control. (c) Gray scale analysis of TNF-α, IL-12, IL-10, and Arg-1 protein expression levels in SK-N-MC + U937 and SK-N-MC + HuD + U937 groups (‘***’ represents an extremely significant difference [*P* < 0.001]; ‘**’ represents a significant difference [*P* < 0.01]; ‘*’ represents statistical difference [*P* < 0.05]; ‘ns’ means no significant difference).

## Discussion

More than one neuron autoantibody can be found in almost every type of PNS disease; conversely, each antibody can also be reflected in different PNS diseases. The HuD antibody is closely related to the occurrence and development of PNS [13]. The Hu protein family includes HuA (also known as HuR and ELAVL1), widely expressed protein HuB (also known as ELAVL2), HuC (also known as ELAVL3), and HuD neuron-specific proteins [14], which is a newly discovered neuronal marker and specific antigen related to many neural development processes [15]. It regulates the expression of neuron-specific genes at the transcriptional level and regulates the protein during the embryonic development of neurons that is necessary for the growth, development, and survival of mature neurons [16]. Additionally, the HuD protein can promote neural stem cells’ entrance to the mitotic stage and facilitate the differentiation and maturation of neurons, which makes it an essential protein for the formation of neural processes. The HuD protein of the CNS is mainly distributed in the neocortex, hippocampal CA area, entorhinal cortex, and cerebellar projection neurons, as well as the mitral cell layer and granular cell layer of the olfactory bulb. The HuD antibody was first found in PNS with small cell carcinoma. In recent years, there is evidence that the disorder and mutation of HuD antibodies affect the occurrence of nervous system diseases, neuroendocrine cancer, and other diseases [17]. The discovery of autoantibodies associated with CNS diseases promotes the study of PNS [18]. The syndrome produces autoantibodies together with specific nervous system syndromes and cancers, such as the anti-HuD antibody in SCLC encephalomyelitis and the anti-Yo antibody in cerebellar degeneration of gynecological cancer. These autoantibodies have potential diagnostic and prognostic roles in the early detection of nervous system diseases and cancers [19]. Although the HuD antibody is closely related to the pathogenesis of PNS, its potential mechanism remains unclear [20].

Studies have established the relationship between the peripheral nervous system and the immune system, demonstrated that the nerve fibers were closely associated with various subsets of dendritic cells and macrophages [21]. Meantime, the proliferation of macrophages can directly or indirectly activate the peripheral nervous system [22]. Previous studies have analyzed the role of different proteins and antibodies related to neurons and their potential association mechanism with PNS [23]. In this study, the role of the HuD antibody absorbed into nerve cells was analyzed. The results revealed that its absorption promoted the proliferation of M1 macrophages, and in the presence of the HuD antibody, the apoptosis level of neuroma cells increased significantly when neuroma cells were co-cultured with monocytes. However, the apoptosis rate did not change significantly in the presence of the HuD antibody alone or in co-culture with monocytes. The above results suggest that neuroma cells may stimulate macrophages to secrete related inflammatory factors and promote apoptosis through the HuD antibody. By conducting ELISA, we found that after adding the HuD antibody, the levels of TNF-α and IL-12 secreted by macrophages increased significantly, while the levels of anti-inflammatory factors IL-10 and Arg-1 decreased significantly. This may indicate that the HuD antibody stimulates neuroma cells to induce the polarization of M2 macrophages to the M1 phenotype. Results of qPCR tests and Western blots revealed that compared with neuroma cell culture without the HuD antibody, the expression levels of M1 macrophage-inducible factors TNF-α, IL-12, INF-γ, and TGF-β increased in the presence of the HuD antibody, and the expression levels of M2 macrophage-inducible factors IL-10, IL-4, and Arg-1 decreased. This further suggests that after absorbing the HuD antibody, neuroma cells make M1 macrophages secrete inflammatory factors to stimulate the inflammatory injury of neuroma cells, so as to induce the apoptosis of neuroma cells. To the immune system, other reports have verified the proliferation of B cell, CD4 + T and CD8 + T cells in Hu-antibody associated PNS [24]. Here, we discovered a supplement with the new macrophage approach, which enriched the mechanism of PNS.

The results of this study revealed that cerebellar neuronal cells promoted PNS by absorbing the HuD antibody to induce the proliferation of M1 macrophages and related inflammatory responses. Furthermore, these results provide a reference for the study of tumor immunity and autoimmunity related to PNS and provide a theoretical basis for future treatment and prevention of PNS.

## Conclusion

Patients with PNS was characterized by high level of HuD antibody, as well as aberrant immune microenvironment. To explore in detail the mechanism of the occurrence of the PNS, we co-culture CNS cells and mononuclear macrophage together with or without HuD antibody. We showed that HuD antibody could be absorbed by the neuronal cells and promote macrophage M1 polarization, which secreted the inflammatory factors to reduce the CNS cells. These approach might be a potential mechanism to induce the occurrence of PNS.

## Highlights

1. The HuD antibody can be absorbed by the neuroma cells in the occurrence of PNS.
2. Neuroma cells promote the M1 macrophages when stimulated by HuD antibody.
3. M1 macrophages could damage the neuronal cells by secreting inflammatory factors.
